# Electrolyte Disorders and In-Hospital Mortality during Prolonged Heat Periods: A Cross-Sectional Analysis

**DOI:** 10.1371/journal.pone.0092150

**Published:** 2014-03-20

**Authors:** Carmen A. Pfortmueller, Georg-Christian Funk, Alexander B. Leichtle, Georg M. Fiedler, Christoph Schwarz, Aristomenis K. Exadaktylos, Gregor Lindner

**Affiliations:** 1 Department of Emergency Medicine, Inselspital, University Hospital Bern, Bern, Switzerland; 2 Department of General Internal Medicine, Inselspital, University Hospital Bern, Bern, Switzerland; 3 Department of Respiratory and Critical Care Medicine, Otto Wagner Spital Vienna and Ludwig-Boltzmann Institute for COPD and Respiratory Epidemiology, Vienna, Austria; 4 Center for Laboratory Medicine, Inselspital, University Hospital Bern, Bern, Switzerland; 5 Department of Nephrology, Landeskrankenhaus Steyr, Steyr, Austria; University of São Paulo School of Medicine, Brazil

## Abstract

**Background:**

Heat periods during recent years were associated with excess hospitalization and mortality rates, especially in the elderly. We intended to study whether prolonged warmth/heat periods are associated with an increased prevalence of disorders of serum sodium and potassium and an increased hospital mortality.

**Methods:**

In this cross-sectional analysis all patients admitted to the Department of Emergency Medicine of a large tertiary care facility between January 2009 and December 2010 with measurements of serum sodium were included. Demographic data along with detailed data on diuretic medication, length of hospital stay and hospital mortality were obtained for all patients. Data on daily temperatures (maximum, mean, minimum) and humidity were retrieved by *Meteo Swiss*.

**Results:**

A total of 22.239 patients were included in the study. 5 periods with a temperature exceeding 25°C for 3 to 5 days were noticed and 2 periods with temperatures exceeding 25°C for more than 5 days were noted. Additionally, 2 periods with 3 to 5 days with daily temperatures exceeding 30°C were noted during the study period. We found a significantly increased prevalence of hyponatremia during heat periods. However, in the Cox regression analysis, prolonged heat was not associated with the prevalence of disorders of serum sodium or potassium. Admission during a heat period was an independent predictor for hospital mortality.

**Conclusions:**

Although we found an increased prevalence of hyponatremia during heat periods, no convincing connection could be found for hypernatremia or disorders of serum potassium.

## Introduction

Disorders of serum sodium and potassium are common in hospitalized as well as outpatients with a prevalence of about 15% in emergency patients [1]. Both, hypo- as well as hypernatremia and dyskalemias have been reported to be independent predictors of mortality [1], [2]. In the outpatient setting the etiology of disorders of serum sodium and potassium has been mostly linked to diuretic use [1]. Ambulatory acquired hypernatremia was found to be common in elderly patients and residents of nursing homes and it was concluded that it might be due to insufficient accessibility of free water due to immobility and or a decreased sensation of thirst in these patients [3]–[5].

The impact of temperature extremes on the health of vulnerable patient collectives such as the poor, children or especially the elderly is discussed in the medical literature [6], [7]. Even a relationship between heat periods and mortality in the overall population has been shown previously and the high number of deaths during the heat period in Europe in 2003 especially among the elderly found attention by the popular media [8], [9]. Given the pathophysiology of disorders of serum sodium, but also of serum potassium it is well imaginable that during prolonged periods of extreme temperatures with increased sweating an increase in the prevalence of electrolyte disorders due to dehydration or excess intake of free water, as described in endurance runners can be observed [10], [11]. However, so far no study has investigated the impact of temperature extremes on the prevalence of electrolyte disorders. We wanted to investigate whether periods of temperature extremes are associated with an increased prevalence of electrolyte disorders in patients presenting to the emergency department of a large tertiary care facility and whether there is an association between temperature extremes and in-hospital mortality in patients hospitalized during heat periods.

## Materials and Methods

The study was approved and the need for written informed consent was waived by the local institutional review board, the Ethics Commission of the Canton of Bern, Switzerland.

In this cross-sectional analysis, we included all patients presenting to the Department of Emergency Medicine of the Inselspital, University Hospital Bern between 01 January 2009 and 31 December 2010 with measurement of serum sodium, as ordered by the emergency physician in charge. The decision whether to order serum sodium measurement was at the discretion of the emergency physician. Exclusion criteria was age <16 years.

Serum sodium and potassium were determined by the Center for Laboratory Medicine using the Roche Modular ISE 900, Roche Diagnostics, Basel, Switzerland. Creatinine was determined enzimatically using the Roche Modular P800, Roche Diagnostics, Basel, Switzerland. Hyponatremia was defined as a serum sodium <135 mmol/L and hypernatremia as a serum sodium >145 mmol/L. Stratification of serum sodium disorders into borderline, moderate and severe was performed as published earlier [2]. Hypokalemia was defined as a serum potassium <3.5 mmol/L and hyperkalemia as >4.7 mmol/L according to the reference ranges of the Center for Laboratory Medicine.

Of all administered patients we gathered the following data: age, gender, data on hospitalization including length of hospital stay and in-hospital mortality. Additionally, data regarding current diuretic medication including type of diuretic medication and current daily dose was obtained.

Data on daily temperatures including maximum and mean temperatures from the official weather station in the area of Bern was obtained from Meteo Suisse, the official weather service of the Swiss Confederation. Definition of temperature extremes was performed in accordance with Meteo Suisse due to a lack of an internationally accepted definition: period of warmth: 3–5 days with maximum daily temperatures ≥25°C; prolonged period of warmth: ≥5 days with maximum daily temperatures ≥25°C; heat period: 3–5 days with maximum daily temperatures ≥30°C; heat wave: ≥5 days with maximum daily temperatures ≥30°C. We used baseline characteristics and serum creatinine to calculate the estimated glomerular filtration rate in accordance with the Modified Diet in Renal Disease (MDRD) formula.

### Statistical Analysis

Data are presented as means ± standard deviations, medians with 1^st^ to 3^rd^ quartiles or proportions, as appropriate. Between-group comparisons of categorical variables were performed using χ^2^ test.

In order to describe an association between electrolyte disorders and mortality we used a Cox regression model. Predefined covariates were included in the model. The proportionality assumption was tested using log-log plots or Schoenfeld residuals. A two-sided p-value <0.05 was considered statistically significant. Statistical analysis was performed using SPSS (SPSS for Windows release 15.0, Chicago, IL) and STATA (STATA/MP 10.0, College Station, TX).

## Results

During the study period a total of 22,239 patients received measurement of serum sodium at the Department of Emergency Medicine. Median age was 53 years (35 to 67) and 57% of patients were male. 2.229 patients (10%) were 80 years of age or older. 12.864 (42%) were admitted for medical and 9.375 for a surgical reason. Mean serum creatinine was 82 μmol/L (SD 64) and serum urea was 6.4 mmol/L (SD 5.1). 1.626 patients (7.3%) had a MDRD eGFR between 30 and 60 ml/min and 516 patients (2.3%) had a MDRD eGFR below 30 ml/min. Mean serum osmolality, present for 3.524 patients was 304 mosm/kg (SD 23). 2,514 patients (11%) currently took a diuretic medication on presentation with loop diuretics (torasemide and furosemide) being the most common with 1,427 patients (6.4%) taking them. 1,884 (8.5%) had one, 547 (2.5%) had two, and 83 (0.4%) had three or four different diuretic substances as medication. Table 1 gives an overview on the diuretic substances and dosages taken by patients.

**Table 1:** Overview on the diuretic medications and median doses (quartile 1 and quartile 3).

Diuretic	Number of patients (%)	Median dose
**Torasemide**	1.196 (48)	10 (5 to 20)
**Furosemide**	231 (9)	40 (40 to 80)
**Hydrochlorothiazide**	975 (39)	12.5 (12.5 to 12.5)
**Chlorthalidone**	102 (4)	12.5 (12.5 to 25)
**Butizide**	16 (1)	2.5 (2.5 to 5)
**Amiloride**	97 (4)	5 (2.5 to 5)
**Spironolactone**	403 (16)	25 (25 to 50)
**Eplerenone**	15 (1)	25 (25 to 50)
**Indapamide**	58 (2)	1.5 (1.5 to 1.5)
**Metolazone**	121 (5)	5 (2.5 to 5)
**Acetazolamide**	20 (1)	250 (250 to 500)

During the study period a total of 88 days were noted with a daily maximum temperature exceeding 25°C in the area of Bern, while on 16 days the temperature exceeded 30°C. All of these days occurred in the time period from May to September. 5 periods of a temperature exceeding 25°C for 3 to 5 days were noticed and 2 periods with temperatures exceeding 25°C for more than 5 days were noted. Additionally, 2 periods with 3 to 5 days with daily temperatures exceeding 30°C were noted during the study period. An overview on the mean monthly temperatures and humidity during the study period is given in Tables 2 and 3.

**Table 2:** Overview on monthly mean and mean daily maximum temperatures and humidity as well as prevalence rates of electrolyte disorders according to month of admission.

	January2009	February2009	March2009	April2009	May2009	June2009	July2009	August2009	September2009	October2009	November2009	December2009
**Tmean**	–2.9±3.3	–0.4±2.6	4.1±2.2	10.7±2.2	15.2±3.5	16.5±2.6	18.6±2.7	19.6±2.6	15.2±2	9.2±4.5	6.7±2.4	0.7±3.9
**Tmax**	–0.3±3.6	3.1±3.4	8.4±3.5	16.8±3.8	21.2±4.5	22.2±3.6	24.6±3.6	25.9±4.9	20.5±2.9	13.8±4.9	10.3±2.8	3.1±4.1
**Humidity**	87.8±4.8	82.9±6.4	74.8±11.6	69.8±9.4	71±9.8	71.4±8.7	72.7±6.3	72.4±8.2	79.4±5.2	80.9±6.6	84.8±5.3	85.2±5.6
**Hyponatremia cases**	107	85	98	90	87	87	96	87	93	76	72	85
**Hyponatremia %**	10,2	8,7	9,6	9,2	8,8	9,3	9,6	8,7	10,1	8,2	8,3	9,4
**Hypernatremia cases**	20	2	13	11	12	12	10	14	10	12	15	13
**Hypernatremia %**	2	0,2	1,3	1,1	1,2	1,3	1	1,4	1,1	1,3	1,7	1,4
**Hypokalemia cases**	130	90	104	108	88	84	115	106	120	97	90	106
**Hypokalemia %**	12,4	9,2	10,2	11,1	8,9	8,9	11,5	10,6	13,1	10,4	10,4	11,8
**Hyperkalemia cases**	58	35	64	45	52	34	43	40	34	43	38	35
**Hyperkalemia %**	5,6	3,5	6,3	4,6	5,3	3,6	4,3	4	3,7	4,6	4,4	3,9

While Tmean is mean monthly temperature, Tmax is mean maximum daily temperature and Humidity is mean monthly humidity. Temperatures given in °Celsius and humidity in percent.

**Table 3:** Overview on monthly mean and mean daily maximum temperatures and humidity as well as prevalence rates of electrolyte disorders according to month of admission.

	January2010	February2010	March2010	April2010	May2010	June2010	July2010	August2010	September2010	October2010	November2010	December2010
**Tmean**	–2.0±2.5	0.1±4.3	4.1±5.3	9.7±3.8	11.8±3.3	16.6±3.5	20.3±3.1	17.4±2.9	13.3±2.3	8.6±3.7	4.8±5.2	–1.5±4.0
**Tmax**	0.1±2.8	3.5±4.9	8.9±6.6	15.6±4.8	15.9±4.8	21.8±4.7	26.3±4.4	22.1±4.2	18.7±3.3	12.6±4.4	7.9±6.2	1.6±4.1
**Humidity**	84.9±5.8	78.9±8.7	70.7±9.8	66.2±6.6	76.2±9.4	74.9±8.3	68.8±7.5	75.4±5.9	76.8±4.5	82.8±4.2	83.9±5.3	84.5±6.5
**Hyponatremia cases**	75	67	75	76	88	62	99	76	91	89	60	65
**Hyponatremia %**	8,3	7,8	8,5	8,9	9,9	6,7	10,2	8,3	10,4	9,8	7,2	7,2
**Hypernatremia cases**	22	17	22	15	18	17	9	9	13	18	12	19
**Hypernatremia %**	2,4	2	2,5	1,8	2	1,8	0,9	0,9	1,5	2	1,4	2,1
**Hypokalemia cases**	86	91	88	107	90	122	130	104	117	109	86	91
**Hypokalemia %**	9,5	10,7	10	12,6	10,1	13,3	13,4	11,4	13,4	12	10,3	10,1
**Hyperkalemia cases**	42	33	36	42	40	31	42	38	42	35	32	40
**Hyperkalemia %**	4,6	3,9	4,1	4,9	4,5	3,4	4,3	4,2	4,8	3,9	3,9	4,4

While Tmean is mean monthly temperature, Tmax is mean maximum daily temperature and Humidity is mean monthly humidity. Temperatures given in °Celsius and humidity in percent.

**Figure 1:**
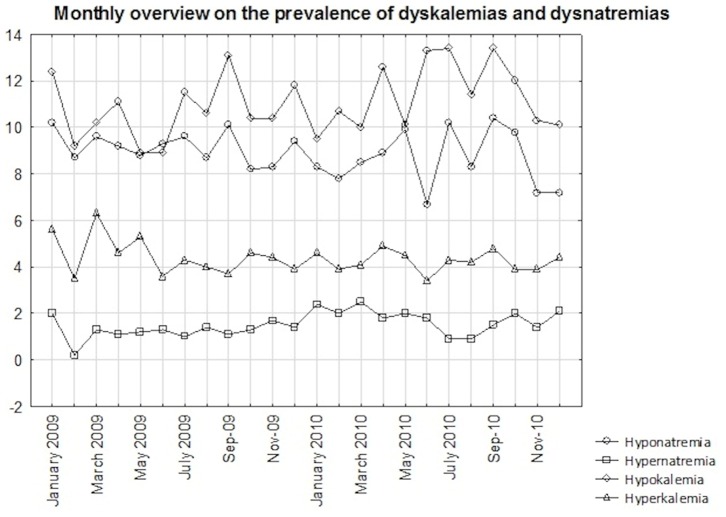
Monthly prevalence of electrolyte disorders. Numbers are given in percent.

Of the 22.239 patients (58%) included in the analysis, a total of 2.459 (11.1%) presented with hypokalemia and 974 (4%) with hyperkalemia. 1.986 (8.9%) patients had hyponatremia defined as a serum sodium <135 mmol/L. 1.337 patients (67% of patients with hyponatremia) had borderline hyponatremia (>130 and <135 mmol/L), 459 (23%) had moderate (>125 and ≤130 mmol/L) and 190 (10%) had severe (≤125 mmol/L) hyponatremia. 335 patients (1.5%) had hypernatremia with a serum sodium exceeding 145 mmol/L. 306 patients had borderline hypernatremia (>145 and ≤150 mmol/L), 21 had moderate (>150 and ≤155 mmol/L) and 8 patients had severe (>155 mmol/L) hypernatremia. The median monthly prevalence of hyponatremia was 8.85% (8.3–9.65), of hypernatremia 1.4% (1.18–2), of hypokalemia 10.65% (10.1–12.1) and of hyperkalemia 4.3% (3.9–4.6). An overview of the monthly prevalences of electrolyte disorders is given in Figure 1. Tables 2 and 3 give the prevalence rates of electrolyte disorders according to the admission month.

There were weak inverse correlations between the daily maximum temperatures and serum sodium (R = −0.04, p<0.05) and serum potassium (R = −0.03, p<0.05) and a positive correlation with serum creatinine (R = 0.01, p<0.05). Also, we saw an inverse correlation with daily sunshine duration with serum sodium (R = −0.02, p<0.05) and serum potassium (R = −0.02, p<0.05). A significant correlation between humidity levels and serum sodium was found (R = 0.02, p<0.05).

Comparing the prevalences of electrolyte disorders of patients admitted during days with a daily maximum temperatures above or equaling 30°C, significantly more patients presented with hyponatremia during hot days (11 versus 9%, p = 0.04. No difference was found for hypernatremia (2.1 versus 1.5%, p = 0.28), hypokalemia (9.8 versus 11%, p = 0.34) and hyperkalemia (4.6 versus 4%, p = 0.76). During heat periods (3 to 5 days with daily maximum temperatures ≥30°C) no difference was found for hyponatremia (8.5 versus 9%, p = 0.54), hypernatremia (1.3 versus 1.5%, p = 0.46), hypokalemia (11.9 versus 11%, p = 0.34) and hyperkalemia (3.7 versus 4%, p = 0.23).

In the group of the elderly patients with an age ≥80 years also no difference between patients admitted during heat periods and those admitted during a time without heat periods was found for hyponatremia (18.3 versus 16.9%, p = 0.66), hypernatremia (0 versus 1.9%, p = 0.09), hypokalemia (10.1 versus 10%, p = 0.83) and hyperkalemia (10.6 versus 10.1%, p = 0.95).

Overall, 588 patients (2.6%) died during hospitalization. Detailed mortality rates for subgroups are given in Table 4.

**Table 4:** Mortality rates overall and in patient subgroups.

Patient group	Died	Survived	Mortality
**Overall**	588	21.651	2.6%
**Age ≥80 years**	141	2.088	6.3%
**Hyponatremia**	130	1.856	6.5%
**Hypernatremia**	26	309	7.8%
**Hypokalemia**	112	2347	4.6%
**Hyperkalemia**	102	862	10.5%

In the multivariate Cox regression model the presence of hyponatremia, hypernatremia, elderly age (≥80 years) as well as admission to the hospital during a heat period were independent predictors of mortality. Details on the results of the Cox regression are given in Table 5.

**Table 5:** Factors associated with mortality in the multivariate Cox regression model.

Factor	Odds ratio	95% confidence interval	Significance
**Serum sodium**			
**Normal serum sodium (reference)**			
**Hyponatremia**	1.72	1.36–2.18	p<0.0001
**Hypernatremia**	3.62	2.34–5.59	p<0.0001
**Age ≥80 years**	1.51	1.04–2.19	p<0.0001
**Admission during heat period**	1.58	1.24–2.0	P = 0.03
**Serum creatinine**	1.002	1.001–1.002	p<0.0001

## Discussion

In the present study we investigated the impact on temperature extremes on the prevalence of electrolyte disorders in patients presenting to the emergency department of a large tertiary care hospital. Although we found weak inverse correlations between daily maximum temperatures and serum sodium and potassium and a significantly higher prevalence of hyponatremia during extremely hot days, a clear link between temperature extremes and the prevalence of dysnatremias and dyskalemias could not be shown. Even in the group of suspectedly most vulnerable patients above age 80 years we could not find a connection between hot periods and an increased prevalence of disorders of serum sodium and potassium in patients presenting to our emergency department. However interestingly we found admission to the hospital during periods of (for the region) extraordinary warmth to be independently associated with mortality.

So far, this is the first study investigating the effect of temperature extremes on the prevalence of dysnatremias and dyskalemias in a large set of outpatients presenting to the emergency department of a large university hospital. The theoretic link between hot temperatures and the development of dysnatremias is obvious: Hot temperatures usually result in increased sweating and loss of hypotonic fluids [12]. In patients with either a disturbed sense of thirst or an impaired access to free water, as for example some patients in nursing homes, this can result in development of dehydration and development of hypernatremia [3], [5], [13]. On the other hand, increased sweating and excess substitution of fluid losses by ingestion of hypotonic fluids has been described in the setting of endurance exercisers [11], [14], [15]. Both mechanisms may also play a role in the development of dyskalemias. However, although we found an increased prevalence of hyponatremia in patients admitted during periods of heat, no such connection could be shown for hypernatremia, hypokalemia or hyperkalemia. Surprisingly, also in elderly patients we did not find a convincing connection between temperature extremes and the prevalence of electrolyte disorders.

On the other hand, our study showed that patients admitted to the hospital during periods of heat had an independently increased mortality compared to those hospitalized during cooler periods. A recent study found that patients with bradyarrythmias with need for transient pacing were significantly more often presenting during the hottest months of the year and many of them showing signs of dehydration [16]. It was found in sports medicine studies that dehydration leads to decreased strength and power [17]. Also, decrements in physical, visuomotor, psychomotor, and cognitive performance were noted in previous studies [18]. Taken together it appears that there are enough reasons to explain an increased mortality in patients exposed to excess temperatures during heat periods. However, although often suspected, based on our current data, electrolyte disorders seem not to play a part.

Our study is limited by some factors: we do not have data on how many of our patients were nursing home residents or had an impaired excess to free water for other reasons. Naturally, we do not have information on the exact temperatures the patients were exposed to before admission due to variances in living conditions (air condition, homes situated at higher sea level, etc.).

## Conclusions

We present the first study investigating the effect of heat periods on the prevalence of dysnatremias and dyskalemias. Although we found an increased prevalence of hyponatremia during heat periods, no convincing connection could be found for hypernatremia or disorders of serum potassium.
